# Synergistic tissue destruction by *Staphylococcus aureus* and *Staphylococcus epidermidis* in a 3D human skin biofilm equivalent

**DOI:** 10.1016/j.bioflm.2026.100361

**Published:** 2026-04-02

**Authors:** Rima Nuwayhid, Nguyen Ngoc-Huyen, Norman Lippmann, Nadine Dietze, Laura Kursawe, Judith Kikhney, Annette Moter, Philipp Kobbe, Frank Siemers, Andreas Roth, Stefan Langer, Christina Pempe, Olga Kurow

**Affiliations:** a Department of Orthopaedic, Trauma and Plastic Surgery, University Hospital Leipzig, 04103, Leipzig, Germany; b Department of Upper Extremity Surgery and Microsurgery, Institute of Traumatology, Orthopedics and Plastic Surgery of Central Hospital 108, Hanoi, Viet Nam; c Institute of Medical Microbiology and Virology, University Hospital Leipzig, 04103, Leipzig, Germany; d MoKi Analytics GmbH, BioCity Leipzig, 04103, Leipzig, Germany; e Moter Diagnostics, BioCity Leipzig, 04103, Leipzig, Germany; f Department of Trauma and Reconstructive Surgery, Martin-Luther-University Halle-Wittenberg, 06120, Halle, Germany; g Department of Trauma and Reconstructive Surgery, BG Klinikum Bergmannstrost, 06112, Halle, Germany; h Department of Plastic, Hand Surgery and Burn Care, BG Klinikum Bergmannstrost, 06112, Halle, Germany

**Keywords:** 3D tissue engineering, Skin model, Biofilm, Polymicrobial infection, Cytokine response, *Staphylococcus aureus*, *Staphylococcus epidermidis*

## Abstract

Staphylococci are common skin commensals that can transition into opportunistic pathogens, particularly in biofilm-associated and polymicrobial infections. However, how interspecies interactions modulate virulence remains poorly understood, partly due to a lack of human-relevant models. We adapted a human cell–based three-dimensional skin equivalent (3DSE) into a biofilm infection model using monospecies biofilms of *Staphylococcus aureus* or *Staphylococcus epidermidis*, and a dualspecies co-culture. Biofilm architecture and spatial distribution were analysed by histology and fluorescence in situ hybridisation, while bacterial dominance was assessed by colony-forming unit counts. Host responses were evaluated using a composite biofilm destruction score, lactate dehydrogenase release, apoptosis and tight junction integrity, and cytokine profiling. The 3DSE supported robust, species-specific biofilm formation. Notably, despite reduced biofilm mass, dualspecies biofilms caused the most severe tissue damage, cytotoxicity and epithelial disruption. Although *S. aureus* dominated in co-culture, pathogenicity was not dependent on bacterial load. These findings demonstrate synergistic host modulation in polymicrobial staphylococcal biofilms and establish the 3DSE as a physiologically relevant platform for studying skin biofilm infections.

## Highlights

• A 3D human skin equivalent models staphylococcal biofilms.
• *S. aureus* and *S. epidermidis* show distinct spatial biofilm formation patterns.
• Co-culture biofilms cause synergistic tissue damage and cytotoxicity.
• Dual-species infection disrupts epithelial integrity beyond mono-species effects.
• The model enables mechanistic study of host–microbe interactions in human skin.

## 1 Introduction

To reflect the high prevalence of the commensal *Staphylococcus epidermidis* on human skin and mucosa, its original nomenclature *Staphylococcus albus* was changed to designate it as the *Staphylococcus* of skin [1,2]. Together with *Staphylococcus aureus* it may colonise skin and nasal cavities without impairment to the host's health [3,4]. But under specific conditions, these seemingly harmless colonisers can become pathogens. *S. epidermidis* is a leading cause of implant-associated infections as well as an important cause of nosocomial bloodstream infections, particularly catheter-related bacteraemia [2]. *S*. *aureus* accounted for more than one million deaths globally in 2019 [3,5]. It belongs to the most common cause of diseases as diverse as infective endocarditis, bone and joint infections, skin and soft tissue infections and also frequently isolated in cases of pneumonia, pleural empyema and infections of medical implants [[6], [7], [8], [9], [10], [11], [12], [13], [14]].

The Janus-faced role of staphylococci as either commensals or opportunistic pathogens can be observed in skin diseases such as atopic dermatitis (AD). Here, *S. aureus* colonisation increases from around 20% in healthy individuals to 100% in severe cases [[15], [16], [17], [18], [19]]. It should be noted, that these frequently cited numbers stem from foundational studies conducted in small cohorts with some of them dating back to the 1970s and 1980s. More recent studies in a larger cohort confirm a correlation of *S. aureus* colonisation density with lesion severity and symptom improvement after therapeutic reduction of *S. aureus* load [20]. While certain *S. epidermidis* strains can inhibit *S. aureus* biofilm formation *in vitro* and have therefore been proposed to exert protective effects, other strains are likewise linked to severe AD and can change the epidermal morphology *in vitro* [[21], [22], [23]]. Thus, staphylococci are a paradoxical group: beneficial or neutral under homeostasis, yet capable of driving pathology when the ecological balance is disturbed.

A key factor in this switch from harmless colonisation to invasive infection may be biofilm formation, which both *S. aureus* and *S. epidermidis* are capable of [24,25]. Biofilms are structured communities of microorganisms characterised by their ability to encase themselves in a self-produced extracellular matrix that acts as a protective barrier against immune clearance [26]. Within these biofilms, bacteria are less susceptible to antibiotic treatment and may exchange antibiotic resistance-transferring plasmids. Biofilm formation may contribute to turning staphylococci from benign commensals to pathogens.

Many severe infections occur under polymicrobial conditions, where multiple bacterial species interact within shared biofilms [[27], [28], [29]]. These interactions can be of competitive or cooperative character and can profoundly impact microbial community structure, virulence and host response [[30], [31], [32], [33]]. Such interactions determine microbial composition and host health. Understanding these dynamics is essential for optimising antimicrobial strategies and answer clinically relevant questions, like whether to target one dominant species or all involved pathogens in polymicrobial infections.

Given the increasing use of medical implants, rising immunosuppressing factors on the patient's side and the threat of antimicrobial resistance, staphylococcal biofilm infections represent a highly relevant, growing clinical challenge [2,34,35]. To address this, knowledge on the pathomechanisms of polymicrobial infections is essential and physiologically relevant experimental platforms are needed. While animal models offer insights into systemic reactions, their translational relevance is limited [36]. In contrast, three-dimensional human cell-based tissue models replicate tissue architecture and provide insights into spatial bacterial colonisation, and interactions between microbial species as well as the host [37].

The aim of this study was to establish and evaluate a human cell-based, three-dimensional skin equivalent (3DSE) as a model for investigations into biofilm formation of *S. aureus* and *S*. *epidermidis*, their interbacterial interactions and their effect on human skin-like tissue.

## 2 Methods

### 2.1 Composition of 3D skin equivalent with biofilm

**Fig. 1:**
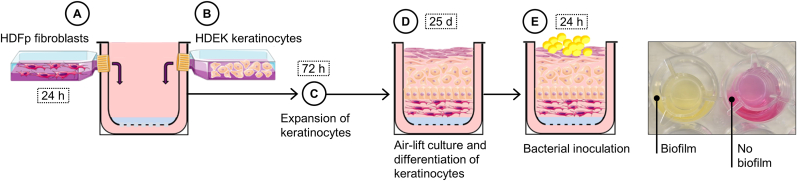
**Schematic overview of the development of the 3D skin equivalent with biofilm.** The model is constructed by co-culturing human fibroblasts (A) to form a dermal layer over 24 h, followed by seeding keratinocytes (B) to generate the epidermal layer. Keratinocytes proliferate to confluence over 72 h (C). The construct is then lifted to the air-liquid interface (D), inducing differentiation and stratification of the epidermal layer over 25 days. The fully stratified 3DSE is subsequently inoculated with a standardised bacterial suspension (E). An incubation period of 24 h allows biofilm formation. The 3D growth medium has a pink colour under physiological conditions; a colour change to yellow indicates a decrease in pH due to bacterial metabolic activity (photograph of 3DSE with and without biofilm, right). *h = hours; d = days.* (For interpretation of the references to colour in this figure legend, the reader is referred to the Web version of this article.)

The fabrication of the 3D skin equivalents (3DSE) was performed as previously described [38]. In summary, 3.5 × 10^4^ primary human dermal fibroblasts (HDFp; pooled; CellnTec Advanced Cell Systems AG, Bern, Switzerland) were embedded in 400 μL of Type I rat-tail collagen gel adjusted to a final concentration of 8.84 mg/mL (10 mg/mL stock; ibidi GmbH, Cat. No. 50201, Gräfelfing, Germany) and placed in 12-well transwell inserts with a polymer mesh support (Fig. 1A). This was followed by incubation and equilibration in fibroblast growth medium at 37 °C, 5% CO_2_ overnight. To improve keratinocyte attachment, the gel surface was coated with 100 μL of fibronectin (Merck, F4759-1 MG; Darmstadt, Germany) at 5 mg/mL in DMEM without additives and incubated at 37 °C for 1 h. Primary human epidermal keratinocytes (PR3D-HPEK-50; CellnTec Advanced Cell Systems AG, Bern, Switzerland; 3.2 × 10^5^ cells per construct) were then seeded onto each gel in growth medium (Fig. 1B). Both fibroblasts and keratinocytes were used at passages 4–8. Adhesion was allowed for 5 h, then culture medium was added to both the apical and basal compartments of the inserts. During 72 h of incubation, keratinocytes proliferate and fully cover the surface (Fig. 1C). When a confluent keratinocyte layer was achieved, the constructs were lifted to allow air contact (Fig. 1D). For 26 days the co-cultures matured with medium changes every three days, yielding fully stratified, organotypic 3D skin equivalents from human cells.

3DSE were divided into four experimental groups: a sterile control or inoculation with either *S. aureus* (ATCC 49230), *S. epidermidis* (PIA 8400) or an equal mixture of both species (Fig. 1E). These clinically relevant staphylococcal species are commonly associated with skin colonisation and biofilm formation. To further assess the interaction dependent behaviour of *S*. *epidermidis,* an additional co-culture group with the less virulent commensal *S*. *S*. *capitis* (DSM 20326) was included for CFU quantification. This design enabled comparison between a highly virulent pathogen (*S. aureus*) and commensal or opportunistic species (*S. epidermidis*), as well as interspecies interactions within dualspecies biofilms. All strains are wild-type clinical isolates. *S. aureus* ATCC 49230 is a well-characterised biofilm-forming reference strain whose virulence repertoire includes cytolytic alpha-toxin, surface adhesins, and immune evasion molecules and is directly relevant to the cytotoxicity and inflammatory responses observed [39]. *S. epidermidis* PIA 8400 was selected for its strong PIA/PNAG-dependent biofilm formation and expresses extracellular serine proteases and phenol-soluble modulins, consistent with the barrier disruption and distinct cytokine profile, particularly elevated MCP-1, observed in this study [40,41]. *S. capitis* DSM 20326 served as a low-virulence comparator sharing biofilm capacity but lacking this broader toxin repertoire.

Bacterial cultures were prepared from overnight-grown colonies, which were harvested and resuspended in sterile phosphate-buffered saline (PBS). Bacterial concentrations were adjusted to 1 × 10^6^ colony-forming units (CFU) per construct. For dualspecies biofilms, equal volumes of each bacterial suspension were combined to achieve a total inoculum of 1 × 10^6^ CFU. The standardised suspensions were applied to the apical surface of fully differentiated 3D skin equivalents. Following inoculation, the constructs were incubated at 37 °C for 24 h under humidified conditions to enable biofilm formation and samples were collected for analysis.

### 2.2 Histology and biofilm score

For histological examination, samples of 3DSE with mature biofilms were fixed in zinc-formaldehyde (Sigma-Aldrich, Taufkirchen, Germany) at 4 °C for 24 h, then embedded in paraffin. Sections were cut using a microtome and mounted onto Superfrost Plus-charged slides (Thermo Fisher Scientific, Waltham, MA, USA). These slides were then deparaffinised in xylene, rehydrated through graded alcohols, and stained with standard haematoxylin and eosin (H&E).

To assess how closely the 3DSE replicates the tissue architecture of human skin, we compared histological images of *ex vivo* skin samples. These were collected from the dorsal hand of a 79-year-old female patient during a surgical procedure at the Department of Plastic Surgery, University Hospital Leipzig, Germany. The study was conducted in accordance with ethical guidelines and approved by the Ethics Committee of the University of Leipzig (approval number 434/20-ek).

In order to quantify biofilm formation and its aggressiveness toward the 3DSE, we used a scoring system developed to incorporate not only the extent of bacterial colonisation but also the degree of tissue destruction. Therefore, the scoring system combines two parameters: the extent of the biofilm and the extent of tissue destruction, with the sum constituting the final biofilm score. To determine the scores, digitised histological sections in H&E staining were analysed using ImageJ software (NIH, Bethesda, MD, USA). The total average skin shown in the histological images was 123,534 μm^2^. The area of the biofilm was measured and expressed as a proportion of the total skin tissue area and graded from 0 to 4. In the same images, the extent of tissue destruction related to invasion depth was graded on the same scale (Table 1). A higher score indicates deeper bacterial invasion and more extensive tissue damage.

**Table 1:** Grading scale for the assessment of biofilm extent and the corresponding degree of tissue destruction.

Biofilm Score				
Extent of Biofilm	Grade	Extent of Tissue Destruction	Grade	Overall Score
No bacteria	0	Intact tissue	0	Sum of Grade ‘Extent of Biofilm’ and Grade ‘Extent of Tissue Destruction’
Planktonic bacteria or immature biofilm	1	Mild destruction with dissolution of the cell junctions in the stratum corneum	1	
Biofilm area corresponding to less than 20% of the skin area	2	Moderate destruction with dissolution of the cell junctions of the cellular epidermal component	2	
Biofilm area corresponding to 20% to 49% of the skin area	3	Pronounced destruction with loosening of the cell junctions of the dermal component	3	
Biofilm area corresponding to 50% or more of the skin area	4	Severe destruction with complete loss of tissue architecture	4	

### 2.3 CFU count

To determine species dominance in the two experimental groups containing two-species biofilms (*S. aureus* + *S. epidermidis* or *S. epidermidis* + *S. capitis*), colony-forming units (CFU) were quantified by serial dilution plating. The complete content of a well was transferred to 500 μl PBS (Gibco, Thermo Fisher Scientific, Waltham, MA, USA) and mechanically disrupted with a sterile mortar. This bacterial suspension was then serially diluted 10-fold. From each dilution, 100 μl was spread onto tryptic soy agar plates (Gibco, Thermo Fisher Scientific, Waltham, MA, USA) in triplicate and the plates incubated at 37 °C for 24 h. Colonies were distinguished based on macroscopic morphology on tryptic soy agar plates. *Staphylococcus aureus* colonies were identified by their golden-yellow colour and smooth, circular appearance, while *Staphylococcus epidermidis* colonies appeared white to off-white, smaller, and slightly domed. *Staphylococcus capitis* colonies were similar in size to *Staphylococcus epidermidis*, but slightly more opaque and glossy, allowing differentiation under careful observation. Colony counts for each species were recorded separately and multiplied with the corresponding dilution factor, resulting in CFU/ml.

### 2.4 Measurement of cytolysis

3DSE were inoculated with the described bacterial suspensions. Following 24 h of incubation at 37 °C, supernatants were collected from both inoculated cultures as well as sterile controls. Lactate dehydrogenase (LDH) release as an indicator of cell membrane disruption was then measured using a Cytotoxicity Detection Kit (Merck KGaA, Darmstadt, Germany).

### 2.5 Cytokine response

To evaluate the inflammatory response following 24 h of bacterial inoculation, we quantified key pro-inflammatory cytokines in the culture supernatants of matured 3DSE. Cytokine levels were measured using DuoSet enzyme-linked immunosorbent assay (ELISA) kits (R&D Systems, Minneapolis, MN, United States) according to the manufacturer's protocols, the cytokines and catalogue numbers are listed in Table 2.

**Table 2:** Commercial ELISA kits used for the quantification of cytokine concentrations.

Cytokine	ELISA kit catalogue number
Interleukin-1α (IL-1α/IL-1F1)	DY200-05
Interleukin-6 (IL-6)	DY206-05
Tumour Necrosis Factor-α (TNF-α)	DY210-05
Interleukin-8 (IL-8/CXCL8)	DY208-05
Interleukin-33 (IL-33)	DY3625B-05
Monocyte Chemoattractant Protein-1 (MCP-1/CCL2)	DY279-05

### 2.6 Immunofluorescence

For the immunohistochemical experiments, tissue sections were prepared as described in the Histology section. The peroxidase activity was inactivated by incubating slides in 1% hydrogen peroxide/methanol for 10 min. Antigen retrieval was performed by heat-mediated epitope unmasking in 0.01 M sodium citrate buffer (pH 6.0) for 10 min. Non-specific binding sites were blocked with 10% normal goat serum (Vector Laboratories, Burlingame, CA, USA; Cat# S-1000) in PBS (0.1 M, pH 7.4) for 1 h. Primary antibodies against Apoptosis-inducing factor (AIF) (Proteintech Group, Rosemont, IL, USA; Cat# 17984-1-AP; 1:400) and ZO-1 (Proteintech Group; Cat# 21773-1-AP; 1:250) were diluted in blocking buffer and applied overnight at 4 °C. Samples were then washed three times for 10 min in Tris-buffered saline (TBS) and were incubated with goat anti-rabbit Alexa Fluor 555-labelled secondary antibodies (Cell Signaling Technology, Danvers, MA, USA; Cat# 4413S; 1:1000). After another set of three 10-min washes in TBS, slides were mounted with DAPI-containing medium (Vector Laboratories, Burlingame, CA, United States) for nuclear counterstaining. The negative controls were processed identically, but without the primary antibodies. Fluorescence imaging was performed with Leica Axiovert 100 microscope (Leica Microsystems GmbH, Wetzlar, Germany) with a Leica digital camera. Analysis of staining localisation and intensity was performed with the software ZEISS ZEN Blue 3.11 (Carl Zeiss Microscopy GmbH, Jena, Germany).

### 2.7 FISH

For fluorescence in situ hybridisation (FISH), 3DSE samples from the monospecies *S. aureus* or *S. epidermidis* groups were fixed using FISHopt® solution (MoKi Analytics, Leipzig, Germany) and subsequently embedded in a cold-polymerising resin, following the supplier's protocol. From these, 2 μm sections were prepared and subjected to FISH, as outlined in a previous report [42]. Briefly, tissue sections were hybridised with a hybridisation buffer containing the nucleic acid stain 4,6-diamidino-2-phenylindole (DAPI) and the FISH probe EUB338 (which detects most bacterial species) and non-EUB338 (to rule out non-specific probe binding), which were labelled at the 5′-end with the indocarbocyanine dye Cy3 or Cy5, respectively. The presence of monospecies colonisation of staphylococci or *S. aureus* was confirmed in selected samples using the *Staphylococcus* genus specific, Cy3-labelled probe STAPHY and the Cy5-labelled *S. aureus* specific probe SAU. All samples were examined with a Zeiss AxioImagerZ2 epifluorescence microscope (Carl Zeiss Microscopy GmbH, Jena, Germany), which was equipped with narrow band filters (AHF Analysentechnik, Tübingen, Germany) and controlled by ZEN Blue software (Carl Zeiss Microscopy GmbH, Jena, Germany).

### 2.8 Statistical analysis

To ensure biological relevance, a minimum of three samples, each derived from a different well, were analysed per experimental group. Each experiment was performed three independent times, resulting in a minimum of nine samples per group for LDH release, cytokine measurement and CFU count. Following exclusion of suboptimal quality stainings, at least seven validated samples per group were included in the histological and immunofluorescence analyses. Quantitative data are presented as means ± standard deviations. For comparisons involving more than two groups, statistical analysis was performed using one-way analysis of variance (ANOVA) followed by Turkey's multiple comparisons test. Where appropriate, comparisons against a single control group were performed using Dunnett's multiple comparisons test. Biofilm Score data were analysed using the Mann-Whitney-U-Test. All statistical analyses were conductedin GraphPad Prism 8.4.3 (GraphPad Software, United States); with p-values <0.05 considered statistically significant.

## 3 Results

### 3.1 Species-specific biofilm architectures with enhanced tissue destruction in dual-species infection

**Fig. 2:**
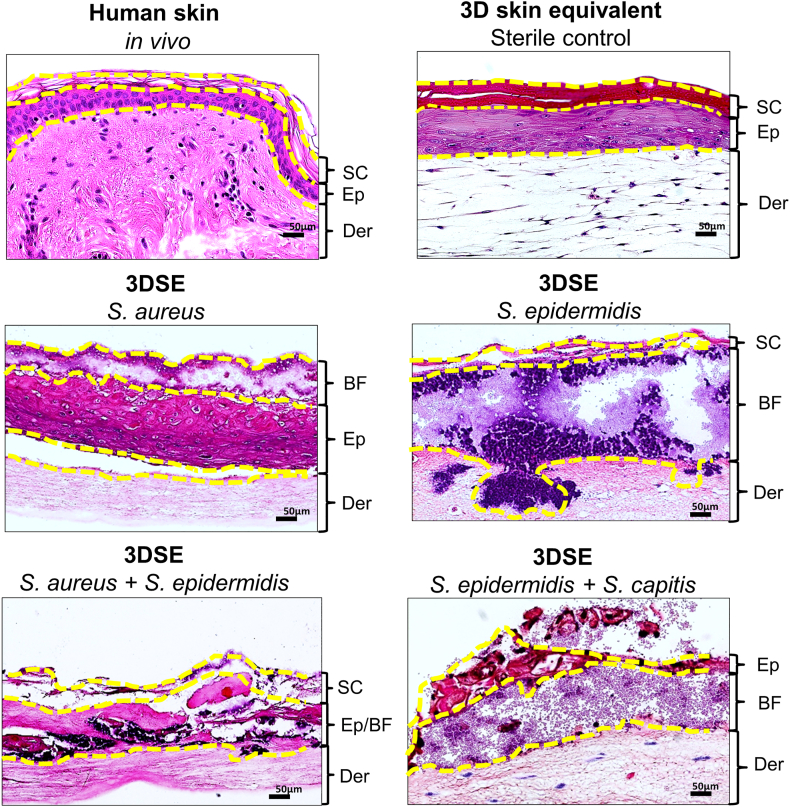
**H&E-stained sections of native human skin and a 3D skin equivalent (3DSE).** The sterile 3DSE control recapitulates the stratified architecture of native skin (upper row). When inoculated with monospecies (middle row) or dualspecies (lower row) bacterial suspensions, dense bacterial clusters are formed. Yellow lines demarcate tissue stratification: *SC = Stratum corneum, Ep = epidermis, Der = dermis, BF = biofilm*. Scale bars: 50 μm. (For interpretation of the references to colour in this figure legend, the reader is referred to the Web version of this article.)

To assess biofilm formation, spatial distribution, and the extent of tissue damage, histological analysis and a composite biofilm scoring system were applied. Control 3DSE maintained in sterile medium exhibited a fully intact tissue architecture equivalent to human skin, consisting of an epidermal component of the *stratum corneum*, *stratum granulosum*, *stratum spinosum*, and *stratum basale* atop a dermal component containing spindle-shaped fibroblasts (Fig. 2). Twenty-four hours after application of the bacterial suspensions to the apical side of 30 day matured 3DSE, biofilm formation was histologically confirmed (Fig. 2).

**Fig. 3:**
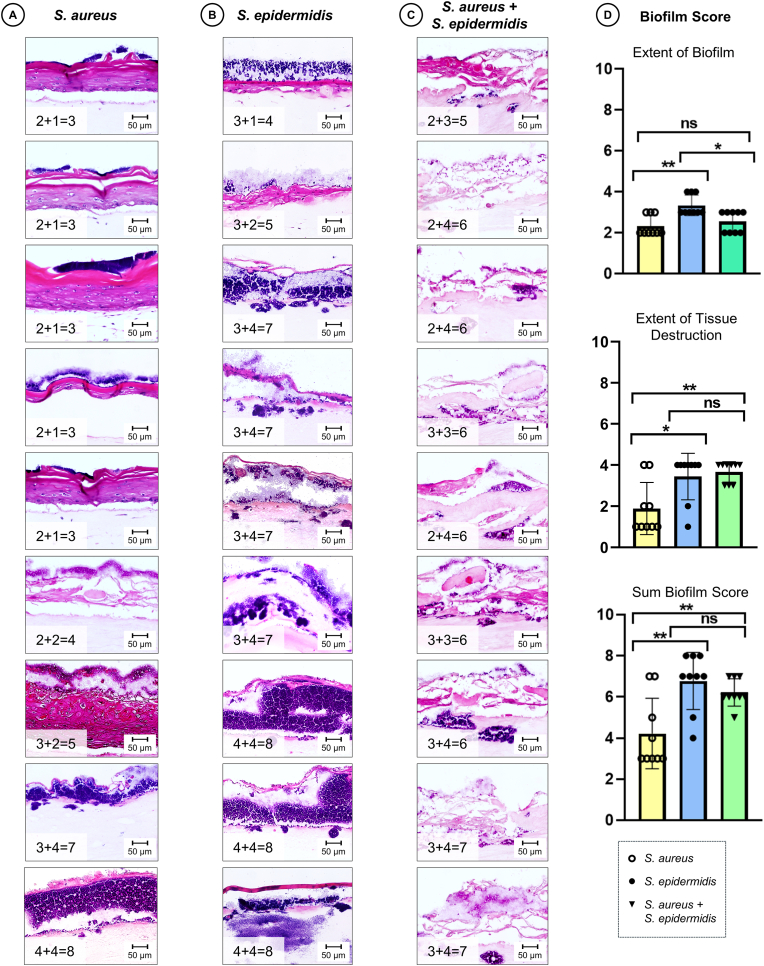
**Histological evaluation and scoring of staphylococcal biofilm formation.** H&E stainings of 3DSE inoculated with *S. aureus* (A), *S. epidermidis* (B) or both (C) for 24 h, demonstrating various levels of biofilm formation and associated tissue destruction. The calculation for the Biofilm Score is overlaid on each image and represents the sum of two parameters: the extent of biofilm formation and the degree of tissue destruction (e.g. “1 + 2 = 3”). Individual grades correspond to the scoring criteria defined in Table 1. Scale bars: 50 μm. (D) Bar graphs depict the total Biofilm Score and the scores for its two subcomponents: the extent of biofilm coverage and the extent of tissue destruction. Results are shown as median ± standard deviation (n = 9 per group). Statistical significance was determined by Mann-Whitney-U-Test; ns: not significant, ∗p < 0.05, ∗∗p < 0.01.

Histological examination revealed distinct pathogen-specific patterns of bacterial spread and biofilm formation. *S. aureus* formed dense aggregates adhering to the epidermal surface, producing focal damage to the stratum corneum (Fig. 3A). In contrast, *S. epidermidis* biofilms penetrated deeper into the 3D tissue, with biofilm localised predominantly at the dermal-epidermal junction and disruption of the *stratum basale* (Fig. 3B). The histological findings in the dualspecies samples, however, showed less pronounced biofilm formation, but advanced tissue destruction. Bacterial colonies had infiltrated the dermal layer in most samples, and the epidermis was disrupted, with its stratification no longer discernible (Fig. 3C). These observations were also reflected in the biofilm score (Fig. 3D). *S. aureus* mono-infections produced the least biofilm (median absolute biofilm area 17.477 μm^2^; median grade extent of biofilm [IQR] = 2 [1]). *S. epidermidis* produced the most biofilm (median absolute biofilm area 42.740 μm^2^; median grade extent of biofilm [IQR] = 3 [1]). The dualspecies cultures developed intermediate biofilm surfaces (median absolute biofilm area 24.241 μm^2^; median grade extent of biofilm [IQR] = 3 [1]). In contrast, significant differences in the capacity for tissue destruction was observed. *S. aureus* resulted in less tissue damage (median grade extent of tissue destruction [IQR] = 1 [1]) compared to *S. epidermidis* (*p* = 0.0345) and the dualspecies biofilm (*p* = 0.0049), which both demonstrated comparable and high levels of destruction (median grade extent of tissue destruction = 4 for both). The tissue damage caused by *S*. *aureus* was remarkably heterogeneous (IQR = 3.0), whereas *S. epidermidis* consistently induced severe tissue damage (IQR = 0.75). These differences in pathogenicity were captured in the overall biofilm score with the median score for *S*. *aureus* (median biofilm score [IQR] = 3 [2]) being significantly lower than that for both *S. epidermidis* (median biofilm score [IQR] = 7 [1]; *p* = 0.0056) and the dualspecies biofilm (median biofilm score [IQR] = 6 [1]; *p* = 0.0265).

**Fig. 4:**
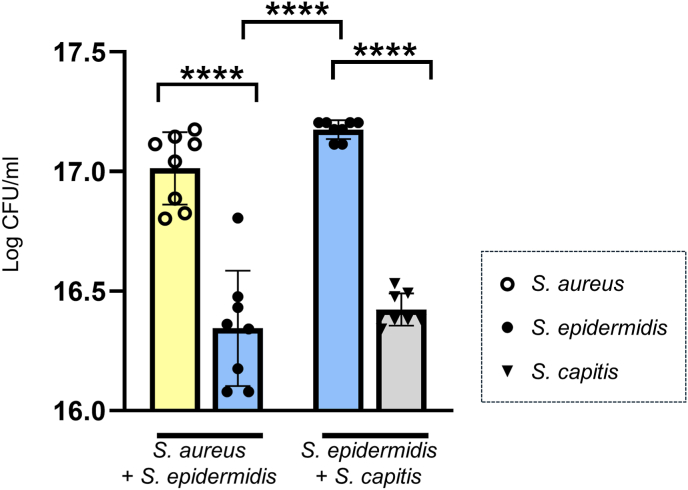
**Colony-forming unit (CFU) count from 3DSE inoculated with two different combinations of dualspecies inoculum.** Data are presented as log-transformed values to account for differences in variances. While *S. epidermidis* growth was suppressed when combined with *S. aureus*, it reached high numbers of bacteria when combined with *S. capitis*. All measurements were independently performed in triplicate, each using three biological replicates (n = 3) per group, resulting in a total of six replicates (n = 9) per group across experiments. Data are presented as mean ± SD. Statistical significance was determined by one-way ANOVA and Turkey's multiple comparisons test; ∗∗∗∗p < 0.0001.

Species dominance is interaction-dependent, with *S. aureus* outcompeting *S. epidermidis*To determine species dominance within polymicrobial biofilms, bacterial load was quantified by colony-forming unit (CFU) analysis. CFU counts revealed shifts in species dominance that was dependent on the combination of bacteria (one-way ANOVA, p < 0.0001). Although the starting inoculum contained equal concentrations of both bacteria, post hoc analysis using Turkey's multiple comparisons test showed that *S*. *aureus* significantly outcompeted *S. epidermidis* (1.0 × 10^17^ CFU/ml vs. 2.2 × 10^16^ CFU/ml; p = 0.0001) (Fig. 4). To assess how *S. epidermidis* behaves in co-culture with a less virulent staphylococcal species, an additional dualspecies condition with *S. capitis* was analysed. Conversely, *S. epidermidis* presented a growth advantage over *S. capitis* (1.5 × 10^17^ CFU/ml vs. 2.6 × 10^16^ CFU/ml; p < 0.0001). In co-culture with *S. capitis, S*. *epidermidis* achieved a significantly higher bacterial load than in co-culture with *S*. *aureus* (p < 0.0001) and no significant difference to the *S. aureus* CFU count.

### 3.2 Dualspecies biofilms induce the highest cytolysis (LDH)

**Fig. 5:**
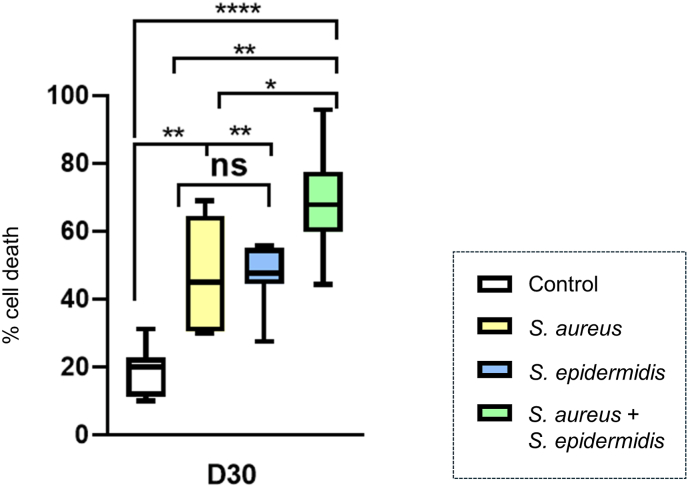
**Quantification of biofilm-induced cytotoxicity via LDH release.** LDH activity was measured in the supernatant of 3DSE following inoculation with sterile control, *S. aureus* monospecies, *S. epidermidis* monospecies, or *S. aureus* + *S. epidermidis* dualspecies biofilms. Cytotoxicity was highest in the dualspecies group. Data are mean ± SD (n = 9, combined from three independent experiments). Statistical significance was determined by one-way ANOVA and Dunnett's multiple comparisons test; ns: not significant, ∗p < 0.05, ∗∗p < 0.01, ∗∗∗∗p < 0.0001.

To quantify biofilm-induced cytotoxicity, cell membrane damage was assessed by measuring LDH release and demonstrated significant differences between groups (one-way ANOVA, p < 0.001). The baseline level of LDH release measured in the NaCl-treated control group was 18.9% and Dunnett's multiple comparisons test revealed that all infected conditions exhibited significantly increased LDH release compared to the untreated control with the highest levels observed in the dualspecies biofilm (*S. aureus* 44.0%, *S. epidermidis* 46.6%, dualspecies 68.9%; all p ≤ 0.004; Fig. 5). However, the difference between the two monospecies groups was not statistically significant. It should be noted that LDH release upon bacterial lysis of staphylococci cannot be excluded and thus a proportion of the measured LDH activity may originate from bacterial instead of human skin cells.

### 3.3 Dualspecies biofilms modulate inflammatory responses

**Fig. 6:**
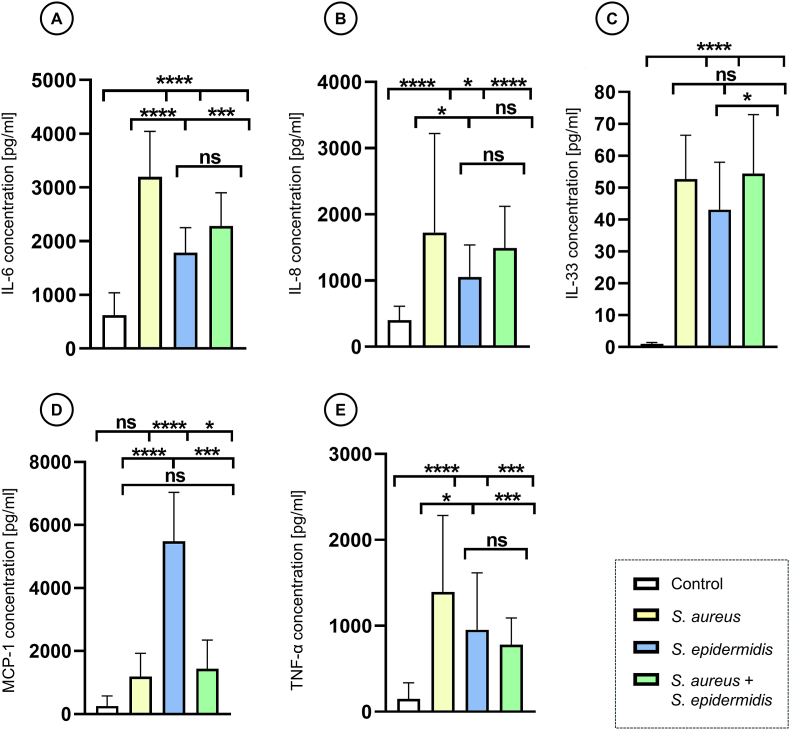
**Quantification of pro-inflammatory cytokines.** Concentrations of (A) interleukin-6 (IL-6), (B) interleukin-8 (IL-8), (C) interleukin-33 (IL-33), (D) Monocyte chemotactic protein-1 (MCP-1) and (E) tumour necrosis factor-alpha (TNF-α) measured via ELISA in culture supernatants of 3DSE sterile control and inoculated with mono- or dualspecies biofilm. Bars represent mean cytokine concentration ± standard deviation. All measurements were independently performed in triplicate, each using three biological replicates (n = 3) per group, resulting in a total of nine replicates (n = 9) per group across experiments. Statistical significance was determined by one-way ANOVA and Dunnett's multiple comparisons test; ns: not significant, ∗p < 0.05, ∗∗∗p < 0.001, ∗∗∗∗p < 0.0001.

To characterise the host inflammatory response to biofilm infection, pro-inflammatory cytokine levels were quantified in culture supernatants. ELISA analysis of cytokines differed significantly between groups (one-way ANOVA, p < 0.0001) and revealed a clear difference between sterile control and biofilm groups, as all cytokines measured were significantly elevated in biofilm samples compared with control except for MCP-1 in *S. aureus* monoculture (Fig. 6). *S. aureus* induced a strong inflammatory response, with significantly higher levels of IL-6, IL-8 and TNF-α than *S. epidermidis* (Dunnett's multiple comparisons test, all p ≤ 0.03). No significant difference was found for IL-33 between *S. aureus* and *S. epidermidis*. *S. epidermidis* showed levels of IL-6, IL-8 and TNF-α comparable to the dualspecies group, with a weaker reaction only in IL-33 (p < 0.04). Remarkably, *S. epidermidis* elicited the strongest expression of MCP-1 with a tenfold increase compared to *S*. *aureus* and the dualspecies biofilm (all p ≤ 0,0006). In the dualspecies biofilm, IL-6 and TNF-α levels were reduced compared to *S. aureus* (all p ≤ 0.0008), but, IL-8-, IL-33- and MCP-1 levels showed no significant differences.

### 3.4 Dualspecies biofilms significantly increase apoptosis (AIF)

**Fig. 7:**
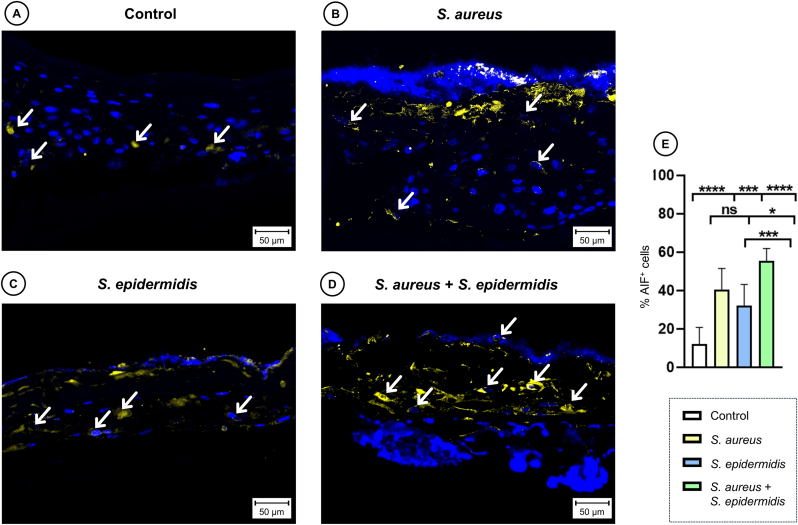
**Immunofluorescence staining for non-caspase dependent apoptosis.** Representative images show expression of apoptosis-inducing factor (AIF, yellow) in sections of 3DSE with sterile control (A) or inoculated with *S. aureus* (B) or *S. epidermidis* (C) monospecies solution or a combined dualspecies inoculum (D). White arrows indicate positively stained cells. All histological examinations were independently performed in triplicate, each using three biological replicates (n = 3) per group. After exclusion of samples of suboptimal quality, a minimum of seven (n = 7) valid samples per group was included in each analysis. Scale bars: 50 μm. (E) Quantitative analysis of immunofluorescence staining for AIF, bar graphs represent the percentage of cells stained positively for AIF. Data are expressed as mean ± SD from at least seven (n = 7) experiments per group. Statistical significance was determined by one-way ANOVA and Tukey's multiple comparisons test; ns: not significant, ∗p < 0.05, ∗∗∗p < 0.001, ∗∗∗∗p < 0.0001. (For interpretation of the references to colour in this figure legend, the reader is referred to the Web version of this article.)

To complement LDH analysis, apoptosis was assessed by AIF staining in control, monospecies, and dualspecies biofilms. Apoptosis levels differed significantly between groups (one-way ANOVA, p < 0.0001). All bacterial inoculation significantly increased AIF-induced apoptosis. No difference in apoptosis rate was discernible between *S. aureus* and *S. epidermidis* mono-species biofilm (Fig. 7). In contrast, the dualspecies biofilm induced significantly higher levels of apoptosis than *S*. *aureus* alone (*p* = 0.019), with an even more pronounced difference to *S. epidermidis* alone (*p* = 0.0006).

### 3.5 Dualspecies biofilms cause the strongest disruption of epithelial cell-cell contacts

**Fig. 8:**
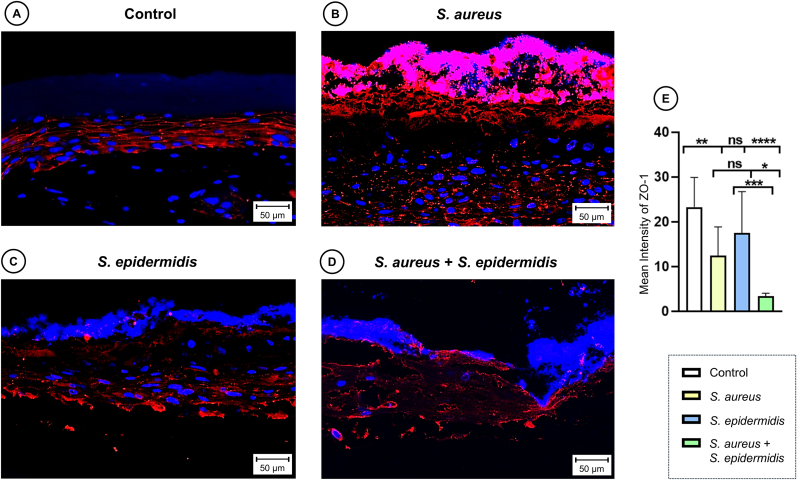
**Immunofluorescence staining for cell contacts.** Representative images show expression of cell–cell adhesion protein ZO-1 (red) in sections of 3DSE with sterile control (A) or inoculated with *S. aureus* (B) or *S. epidermidis* (C) mono-species solution or a combined dualspecies inoculum (D). All histological examinations were independently performed in triplicate, each using three biological replicates (n = 3) per group. After exclusion of samples of suboptimal quality, a minimum of seven (n = 7) valid samples per group was included in each analysis. Scale bars: 50 μm. (E) Quantitative analysis of immunofluorescence staining for ZO-1, bar graphs represent the mean intensity. Data are expressed as mean ± SD from at least seven (n = 7) experiments per group. Statistical significance was determined by one-way ANOVA and Tukey's multiple comparisons test; ns: not significant, ∗p < 0.05, ∗∗p < 0.01, ∗∗∗p < 0.001, ∗∗∗∗p < 0.0001. (For interpretation of the references to colour in this figure legend, the reader is referred to the Web version of this article.)

To evaluate the impact of biofilm formation on epithelial barrier integrity, cell-cell contacts were quantified with, numbers differing significantly between groups (one-way ANOVA, p < 0.0001). Biofilm formation by *S. aureus* significantly reduced the number of cell-cell contacts compared to the control (Turkey's multiple comparison test, p < 0.004; Fig. 8). The most substantial decrease was observed in the dualspecies biofilm, which exhibited a nearly 8-fold reduction relative to the control (Turkey's multiple comparison test, p < 0.0001). Although *S. epidermidis* mono-species biofilms also showed a trend toward fewer contacts, this difference did not reach statistical significance. Similarly, the difference in contact reduction between *S. aureus* and *S. epidermidis* mono-species biofilms was not statistically significant.

### 3.6 FISH confirms structured biofilms and species-specific spatial distribution

**Fig. 9:**
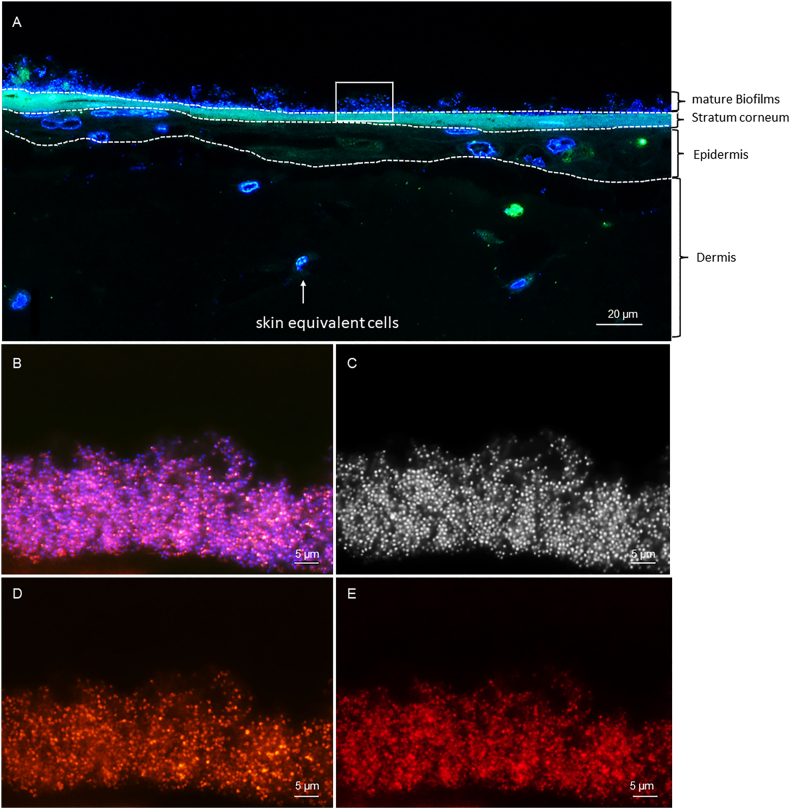
**Representative FISH images of 3DSE colonised with *S. aureus*.** (A) Overview of the 3DSE, a biofilm is present on top of the Stratum corneum. (B) In the inset of panel A, the biofilm is shown at higher magnification (overlay of DAPI and FISH). The same microscopic view as (B) is given in (D-E) with the fluorescence channels separately shown. (C) Dense bacterial cocci stained with DAPI shown in black-and-white. (D) FISH showing hybridisation signals with the Staphylococcus genus-specific probe STAPHY, which is identical to (E) *S. aureus* species-specific probe confirming monospecies colonisation.

**Fig. 10:**
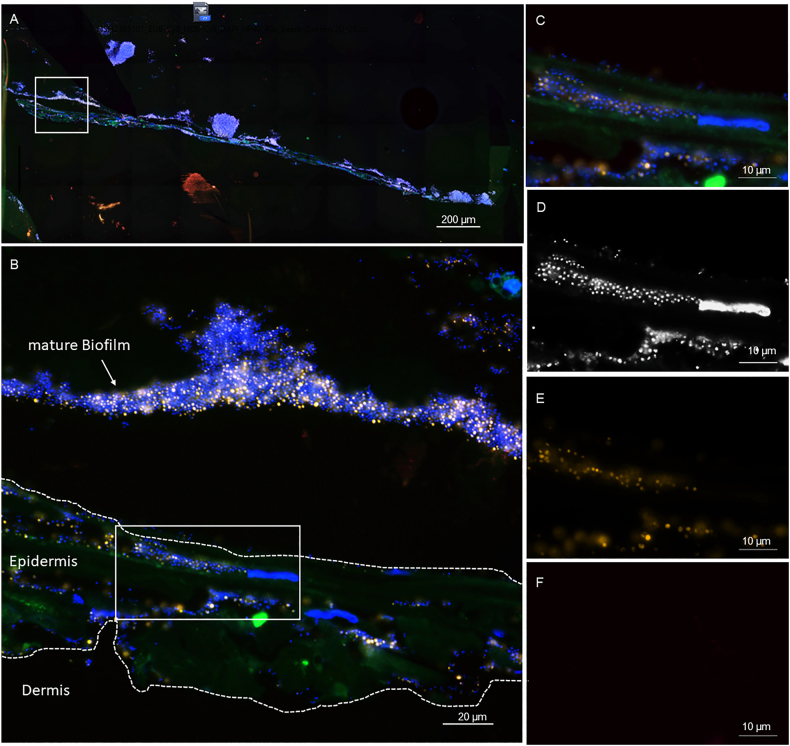
**Representative FISH images of 3DSE colonised with *S. epidermidis*.** (A) Overview of the 3DSE, (B) In the inset of panel A, a biofilm is observed on the surface of the 3DSE with the bacteria infiltrating into the deeper tissue layers. (C) Inset of panel B shows the biofilm at higher magnification. Panels (D–F) show the identical microscopic field as C with the fluorescence channels separately demonstrated. (D) Dense cocci stained with DAPI. (E) FISH showing the hybridisation signals with the Staphylococcus genus-specific probe STAPHY. (F) No signal was observed with the *S. aureus species*-specific probe, indicating monospecies colonisation with coagulase-negative staphylococci.

To confirm mature biofilm presence, and species-specific localisation within the tissue, fluorescence in situ hybridisation (FISH) was performed. FISH confirmed the presence of mature and structured biofilms in the 3DSE samples (Fig. 9, Fig. 10). Staining with DAPI revealed dense bacterial aggregates embedded within the tissue surface, consistent with biofilm architecture. Hybridisation with the Cy3 labelled EUB338 probe showed metabolically active bacteria within these aggregates, based on the ribosome content. The absence of signal in the non-EUB338 control probe ruled out unspecific probe binding. Consistent with histological findings, the *S. aureus* sample presented compact clusters primarily located on the intact stratum corneum (Fig. 9), while *S. epidermidis* demonstrated more dispersed, infiltrative growth (Fig. 10). Specific FISH probes (STAPHY and SAU) confirmed the respective monospecies colonisation.

## 4 Discussion

Based on the results of this study, the 3DSE model successfully recapitulates key aspects of staphylococcal biofilm-associated skin infections and provides a platform for investigating interspecies interactions with human-derived skin tissue.

The staphylococci established distinct species-dependent spatial biofilm distribution patterns in the 3DSE model: *S. aureus* biofilms were compact, superficial and restricted to the *stratum corneum* or formation of microabscesses. *S. epidermidis* formed biofilms more pronounced at the junction of dermal and epidermal layer with deeper penetration and structural disintegration of basal and dermal layer. These observations were confirmed in the histological biofilm scoring, with *S. epidermidis* achieving the highest overall score, indicating extensive biofilm formation and tissue destruction. This illustrates the invasive potential of *S. epidermidis* in biofilm mode, supporting its dual role as both a commensal and a covert pathogen with adhesive proteins facilitating attachment as a crucial first step in biofilm formation [43,44].

It should be noted, that the pronounced tissue destruction caused by *S. epidermidis* in the model appears at odds with its commensal role on healthy skin. This observation reflects the virulence potential of this specific strongly biofilm-forming strain (PIA 8400) in the absence of the resident immune surveillance like Langerhans cells, macrophages and neutrophils, that would normally constrain it in healthy skin [45]. This scenario can be interpreted as analogous to immunocompromised skin.

To provide a comprehensive assessment of microbial pathogenicity in the 3D tissue model, including the spatial distribution, a semi-quantitative scoring system from our group's previous 3D pleural model was adapted, integrating skin invasion depth and tissue destruction in a Gleason-inspired composite score [46,47]. While observer subjectivity and the two-dimensional nature of histological sections are inherent limitations, the system provides a practical and comparable measure of biofilm aggressiveness.

The most striking finding of this study was that dualspecies biofilm caused the most severe tissue damage across all endpoints, despite forming less extensive biofilm mass than either monospecies. Co-culture of *S. aureus* and *S. epidermidis* provoked significantly higher cytotoxicity, increased caspase-independent apoptosis and the greatest disruption of epithelial integrity. Elevated LDH levels and increased AIF expression indicated parallel activation of necrotic and apoptotic pathways. Although partial LDH release from staphylococcal lysis cannot be excluded, the concordant increase in AIF-mediated apoptosis, which exclusively reflects host cell death, supports the conclusion that the observed cytotoxicity primarily arose from host tissue damage [48]. The reduction in cell-cell contacts and extensive histological destruction further underscore this synergistic pathogenic potential. These findings demonstrate that the destructive capacity of polymicrobial staphylococcal biofilms is grossly underestimated when studied in monospecies models [49].

This synergistic virulence likely results from complex pathogen interplay: The vast majority of wildtype *S. epidermidis* express extracellular serine proteases, that inhibit the biofilm formation of *S. aureus*, which potentially leads to more planktonic, invasive *S. aureus* phenotypes with increased tissue penetration [21,50]. Simultaneously, *S. aureus* toxins and *S. epidermidis* proteases, which have been documented for closely related clinical isolates of this species, may cooperatively disrupt the tissue barrier, as evidenced by the reduction of ZO-1-expression and increased cell death marked by elevated levels of LDH and AIF [40,51]. The resulting barrier breach then allows deeper bacterial penetration and consequently host cell death, consistent with clinical observation, that commensal bacteria as part of a polymicrobial infection can worsen clinical outcomes by delaying wound healing and amplified inflammation [52].

An important aspect not directly addressed in this study is the composition of the biofilm extracellular matrix, an important modulator of host immune responses. In *S. aureus*, it comprises PIA/PNAG, eDNA, and surface proteins that shape innate signalling and cytokine profiles [24]. The *S. epidermidis* strain used (PIA 8400) is a strong PIA/PNAG producer, associated with resistance to phagocytosis and altered complement activation, potentially contributing to sustained tissue damage [43]. Future work could include matrix-focused analyses (e.g., PNAG staining, eDNA detection with DNase controls, and protein immunolabelling) to link matrix composition to the observed immune effects. [24]. CFU analysis revealed that *S. epidermidis* was numerically suppressed when co-cultured with *S. aureus*, despite equal starting inocula, indicating competitive dominance of *S. aureus*. This observation is consistent with previous reports describing faster growth kinetics and active interference mechanisms of *S. aureus*, including antimicrobial peptide production and nutrient competition [21,50]. Critically, this numerical suppression of *S. epidermidis* did not reduce pathogenicity. Instead, the dualspecies biofilm caused the most damageacross all endpoints. This demonstrates that bacterial burden alone is a poor predictor of virulence in polymicrobial biofilms and suggests, that *S. epidermidis* contributes to pathogenic synergy through functional rather than numerical dominance. The comparison with the *S. epidermidis and S. capitis* co-culture, in which *S. epidermidis* achieved numerical dominance, confirms that competitive outcome is context-dependent, . This model is therefore a suitable platform to investigate how *S. epidermidis* shifts from a subdominant to a dominant state, a key factor in its success as both a commensal and an opportunistic pathogen. These findings align with reports of virulence driven by interspecies interactions rather than additive effects of individual pathogens [32,49].

In healthy skin, commensal staphylococci can suppress the expression of IL-6, IL-8, and TNF-α in host keratinocytes through TLR-pathway and NF-κB modulation [[53], [54], [55]], a balance maintained while bacteria are confined to an intact epidermal layerThe biofilm-induced tissue destruction observed here, evidenced histologically and by ZO-1 disruption, breaches this anatomical boundary, exposing deeper tissue toa high antigenic load of biofilm and driving the elevated pro-inflammatory cytokine levels despite these immunomodulatory effects.Cytokine profiling further differentiated the pathogen-specificity. *S. aureus* elicited the strongest inflammatory response, with IL-6, IL-8, and TNF-α exceeding *S. epidermidis* levels, consistent with reports of *S. aureus* in biofilm increasing IL-6, IL-8 and TNF-α secretion in human keratinocytes even more than in its planktonic condition [56]. The specific chemokine pattern of *S. epidermidis*, marked by disproportionately elevated MCP-1, could reflect the activity of phenol-soluble modulins (PSM), which stimulate keratinocytes to express IL-6, IL-8, TNF-α and MCP-1 and are associated with *S. epidermidis* and atopic dermatitis (AD) [57]. High levels of IL-33 are also consistent with extracellular serine protease (ESP) secretion documented in related strains [51]. This moderate but distinct cytokine profile aligns with the ambivalent role of *S. epidermidis* and its previously demonstrated induction of lower cytokine levels than *S. aureus* [58].

Interestingly, the dualspecies biofilm reproduced the *S. aureus* inflammatory cytokine pattern, but with suppressed IL-6 and TNF-α levels, suggestive of cooperative immune modulation through interspecies interaction rather than mere additive effects. These data can be interpreted as a cooperative strategy to mitigate inflammation that could otherwise impair bacterial growth. Collectively, our findings emphasise, that the pathogenic potential of polymicrobial staphylococcal infection is greater than the mere sum of individual pathogen's virulence would imply. From a clinical standpoint, these results challenge the notion that commensals like *S*. *epidermidis* can be disregarded in therapeutic decisions in case of polymicrobial infections, particularly in scenarios of immunodeficiency. Instead, targeting all involved pathogens should be considered early on; this might be a key step in mitigating synergistic tissue destruction, chronicity of inflammation and development of bacterial resistance mechanisms.

### 4.1 Model validity and limitations

The considerable translational gap of murine models, specifically regarding infection studies, has led to the development of humanised mouse models [36,59]. This highlights the importance of human cell-based models. The adequate representation of human skin tissue architecture of the 3DSE has been demonstrated previously [38]. Its three-dimensional architecture enables the analysis of spatial colonisation patterns, bridging a gap left by conventional disintegration of samples before plating and monolayer cultures. Robust and pathogen-specific biofilm growth prove its ideal conditions for observing interspecies dynamics. It captures critical aspects of human skin physiology including barrier function and host response. Importantly, by demonstrating that studying pathogens in isolation underestimates their destructive potential, the value of this model for polymicrobial infections is underscored.

Beyond dualspecies interactions, the 3DSE model could be extended to study skin microbiota communities, as skin modulating effects of microbial communities differing from single-species effects have been demonstrated [60]. In principle, additional species could be incorporated into our model. Given the pronounced tissue destruction observed in even the dualspecies biofilm, enabled by absence of immune regulation in the model, increasing microbial complexity would require optimisation of inoculum concentration and bacterial load. Establishing conditions that allow stable biofilm formation while preserving tissue integrity will be an essential next step to enable investigations into microbiome communities in this model.

*Ex vivo* skin models preserve the full cellular complexity of skin, including resident immune cells, appendages and vascular structures; 3DSE today do not reach this level of physiology. However, *ex vivo* samples are limited by restricted tissue availability, donor variability and limited experimental flexibility. In contrast, 3DSE provide a controlled and reproducible platform while mimicking key aspects of human skin. They allow the manipulation of experimental parameters, like cellular composition or ECM characteristics. Standardised fabrication of 3DSE reduces donor variability.

Nevertheless, this model has inherent limitations. The cell co-culture lacks the full cellular complexity of skin, including melanocytes, Merkel cells and resident immune cells. Consequently, immune mechanisms such as leukocyte recruitment cannot be fully studied. In the absence of vascularisation, factors influencing biofilm development like nutrient gradients and immune cell trafficking cannot be investigated. Similarly, as the model contains no blood-derived components, *S. aureus* is not exposed to fibrinogen, a key ligand in bloodstream and implant-associated infections; however, this is not considered a limitation in the context of cutaneous biofilm formation, where adhesion is primarily mediated through interactions with keratinocyte-associated and extracellular matrix components [30].

Future developments of this model could address these limitations. Incorporating immune cells such as Langerhans cells, macrophages and neutrophils would provide a more complex image of cytokine signalling and allow deeper investigation of host immune responses, including phagocytosis and immune invasion strategies employed by staphylococci. The integration of endothelial cells or vascular-like structures through microfluidic systems could enable research into the influence of environmental factors such as nutrient availability and immune cell recruitment on biofilm and its antimicrobial tolerance.

While this study has demonstrated that species interaction modulates virulence and host response in a dualspecies context, physiologically *S. aureus* and *S. epidermidis* exist within far more complex communities as part of the skin microbiome. Extending the model to include additional microbial species would therefore greatly enhance its physiological relevance and provide deeper insights into intermicrobial dynamics.

Beyond expanding microbial complexity, future work should also address the ecological state of the bacteria within this model, in particular, whether their phenotypes more closely resemble those found on healthy skin or in AD lesions. Transcriptomic profiling of bacteria recovered from the 3DSE, compared with published signatures from healthy and AD skin, together with proteomic analysis of secreted factors, would clarify this positioning and further illuminate the transition from commensal colonisation to pathological infection.

## 5 Conclusions

This study establishes a 3D human skin equivalent as a physiologically relevant model for the investigation of polymicrobial biofilms. Using *S. aureus* and *S. epidermidis*, we demonstrated, that while each species alone forms distinct biofilm patterns and induces damage to the host, their co-culture amplifies virulence, resulting in increased cell death and barrier disruption. Our findings indicate that the pathogenic potential in polymicrobial infections exceeds the mere sum of individual pathogen effects.

## CRediT authorship contribution statement

**Rima Nuwayhid:** Writing – review & editing, Writing – original draft, Visualization, Validation, Investigation, Formal analysis, Data curation, Conceptualization. **Nguyen Ngoc-Huyen:** Writing – review & editing, Validation, Formal analysis. **Norman Lippmann:** Writing – review & editing, Validation. **Nadine Dietze:** Writing – review & editing, Validation, Conceptualization. **Laura Kursawe:** Writing – review & editing, Visualization, Investigation. **Judith Kikhney:** Writing – review & editing, Visualization, Investigation. **Annette Moter:** Writing – review & editing, Investigation. **Philipp Kobbe:** Writing – review & editing, Resources, Funding acquisition. **Frank Siemers:** Writing – review & editing, Resources, Funding acquisition. **Andreas Roth:** Writing – review & editing, Supervision, Resources, Funding acquisition. **Stefan Langer:** Writing – review & editing, Supervision, Resources, Funding acquisition. **Christina Pempe:** Writing – review & editing, Visualization, Supervision, Project administration, Formal analysis. **Olga Kurow:** Writing – review & editing, Visualization, Supervision, Project administration, Methodology, Funding acquisition, Formal analysis, Data curation, Conceptualization.

## Ethics declarations

The human skin sample was collected from the dorsal hand of a patient undergoing surgery at the Department of Plastic Surgery, University Hospital Leipzig, Germany. Ethical approval was granted by the Ethics Committee of the University of Leipzig, approval number 434/20-ek. The study was conducted in accordance with the local legislation and institutional requirements. The participant provided their written informed consent to participate in this study.

## Declaration of generative AI and AI-assisted technologies in the writing process

During the preparation of this work the authors used Claude (claude.ai, Anthropic PBC, United States) to assist with improving the readability and language of the manuscript. Following the use of this tool, the authors reviewed and edited the content thoroughly. The authors take full responsibility for the content of the published article.

## Funding

This work was supported by 10.13039/501100003417Deutsche Gesetzliche Unfallversicherung (grant number 10.13039/501100003417DGUV FR 335).

## Declaration of competing interest

The authors declare the following financial interests/personal relationships which may be considered as potential competing interests: Olga Kurow reports financial support was provided by German Social Accident Insurance. Rima Nuwayhid reports financial support was provided by German Social Accident Insurance. Judith Kikhney is CEO of MoKi Analytics GmbH. Annette Moter and Judith Kikhney are shareholders of MoKi Analytics GmbH. Annette Moter is the owner of the private practice Moter Diagnostics. The other authors declare that they have no known competing financial interests or personal relationships that could have appeared to influence the work reported in this paper.

## Data Availability

The Data presented in this manuscript are available to the public at https://doi.org/10.6084/m9.figshare.31036939.
